# Nitric Oxide and Proline Modulate Redox Homeostasis and Photosynthetic Metabolism in Wheat Plants under High Temperature Stress Acclimation

**DOI:** 10.3390/plants12061256

**Published:** 2023-03-10

**Authors:** Zebus Sehar, Iqbal R. Mir, Sheen Khan, Asim Masood, Nafees A. Khan

**Affiliations:** Plant Physiology and Biochemistry Laboratory, Department of Botany, Aligarh Muslim University, Aligarh 202002, India; seharzebus5779@gmail.com (Z.S.); m3riqbal@gmail.com (I.R.M.); khansheen192@gmail.com (S.K.); asim.bot@gmail.com (A.M.)

**Keywords:** antioxidant enzymes, nitric oxide, osmolytes, gas exchange, heat stress

## Abstract

The effects of exogenously-sourced NO (nitric oxide, as 100 µM SNP) and proline (50 mM) in the protection of the photosynthetic performance of wheat (*Triticum aestivum* L.) plants against heat stress were investigated. The study focused on the mechanisms of proline accumulation, activity, gene expression of antioxidant enzymes, and NO generation. Plants were exposed to a temperature of 40 °C for 6 h per day over 15 days, then allowed to recover at 28 °C. Heat-stressed plants showed increased oxidative stress, with higher levels of H_2_O_2_ and TBARS (thiobarbituric acid reactive substances) and increased proline accumulation, ACS activity, ethylene evolution, and NO generation, which in turn leads to increased accumulation of antioxidant enzymes and reduced photosynthetic attributes. In the tested wheat cultivar, the exogenous application of SNP and proline under heat stress improved the photosynthesis and reduced oxidative stress by enhancing the enzymatic antioxidant defense system. Potentially, the promoter AOX (alternative oxidase) played a role in maintaining redox homeostasis by lowering H_2_O_2_ and TBARS levels. The genes for GR antioxidant and photosystem II core protein encoding psbA and psbB were highly up-regulated in nitric oxide and proline treated heat-stressed plants, indicating that ethylene positively impacted photosynthesis under high temperature stress. Moreover, nitric oxide supplementation under high temperature stress optimized ethylene levels to regulate the assimilation and metabolism of proline and the antioxidant system, lowering the adverse effects. The study showed that nitric oxide and proline increased high temperature stress tolerance in wheat by increasing the osmolytes accumulation and the antioxidant system, resulting in enhanced photosynthesis.

## 1. Introduction

As has been the case for many years now, anthropogenic activities that are not planned out and deliberate have been the main cause of climate change aggravation, which has become the greatest threat to the environment. It is impossible to avoid the effects of long-term changes in the climate on plants. High temperature stress (HS) is a severe abiotic stressor that limits metabolism, development, and crop output on a global scale [1,2,3]. Many biochemical pathways crucial for plant growth and development are susceptible to damage from heat stress [3]. High temperature stress leads to the generation of excess reactive oxygen species (ROS), which changes the membrane proteins’ composition and activities. Heat stress changes the expression of genes of the antioxidant enzymes APX (ascorbate peroxidase) and GR (glutathione reductase), multiprotein bridging factor 1 C (MBF 1C), and N-acetylcysteine (NAC), which directly protect plants against heat stress [4,5]. Currently, heat stress is a primary concern for crop output; therefore, finding ways to maintain good crop yield under heat stress is a primary agricultural goal. Plants accumulate inorganic solutes such as proline (Pro) to defend themselves under stressful conditions to combat the osmotic crisis [2,3,4,5,6]. Proline synthesis upregulation has been shown to be an adaptive mechanism for stress tolerance [6]. It can help to organize different cellular structures and proteins, maintain cell turgor through osmotic adjustment, and adjust the antioxidant system to restore cellular homeostasis and redox balance [2,3,4,5,6]. For these protective mechanisms to work, intricate regulatory signaling molecules such as different phytohormones are necessary [2,3,4,5,6]. Phytohormones are the primary molecules that coordinate a variety of plant growth and development processes, functioning as chemical messengers [3]. By stimulating stress-responsive regulatory genes related to plant growth, they serve as crucial endogenous signaling molecules that modulate numerous physiological processes in response to heat stress [2,3,4].

Due to their involvement in various physiological, biochemical, and cellular processes, ethylene, nitric oxide, and hydrogen sulfide have gained attention as important gaseous signaling molecules in plants [2,4,7]. The ethylene concentrations are associated with plant responses to growth and developmental problems caused by heat stress [8,9,10]. In addition to this, ethylene signaling improves a plant’s thermo-tolerance by preserving chlorophyll levels and lowering heat-induced oxidative stress [11].

Nitric oxide is receiving attention from plant scientists because of its involvement in plant stress resistance, though its impact on heat stress tolerance remains a subject of debate. As a gaseous free radical molecule, it has been associated with various biological processes in plants [3,4]. Being a small diatomic molecule, nitric oxide’s properties, including its small size, short half-life, lack of charge, and high diffusibility as a chemical messenger in plant signaling contribute to its significance [12]. Over time, it became clear that NO is essential for several plant processes, including seed germination [3,4,13], physiological activities and senescence [14], fruit ripening [15], and plant abiotic stress responses [4,16]. Many researchers have concentrated on describing the pivotal role of NO in regulating different plant hormone-induced growth and stress reactions [6,17,18]. It is crucial to consider NO’s crosstalk since it may function as a shared signaling element for various elicitors and phytohormones, such as ABA (abscisic acid) [19].

In terms of the most significant stable food for the human population, wheat (*Triticum aestivum* L.), belonging to the Poaceae family, comes in second. It accounts for 30% of the global grain production, 50% of the grain trade, and 20% of the world’s per-person calorie intake [20]. Due to its extreme sensitivity to climate change, *T. aestivum* suffers from heat stress in the world’s arid, semiarid, tropical, and subtropical regions, drastically affecting its productivity [21]. Wheat plants respond to heat stress well in the range of 17 to 23 °C [22], but higher temperatures over 30 °C have an adverse effect. It is negatively impacted by heat and drought, and a loss in worldwide wheat yield of 4.1–6.4% by the middle of the twenty-first century is forecasted as a consequence of an increase in global temperature of 1 °C [23]. Heat stress decreases wheat plant growth and seed germination [24], affecting the plants’ photosynthesis by impairing electron transport, turning off the photosystem II center, degrading proteins, and ultimately reducing yield. It is concerning that recent rises in the average global temperature, and the frequency of high temperatures, pose a severe threat to the quality and output of wheat, as wheat is a staple crop that plays a critical role in global food security. Being a heat-susceptible crop, more future research will be necessary to better understand how to mitigate the adverse effects of heat stress on wheat and protect the global food supply. Although there are few publications on the effects of NO and proline on wheat plants’ ability to withstand heat stress, no information is available on the combined role of NO and proline in affecting photosynthesis under heat stress by controlling antioxidant metabolism and ethylene production. It has not been investigated how NO and proline safeguard the photosynthetic pigment system, specifically by regulating the expression of associated genes in wheat leaves during heat stress.

## 2. Results

### 2.1. NO Increases Growth and Photosynthetic Characteristics of Wheat under High Temperature Stress

**Figure 1:**
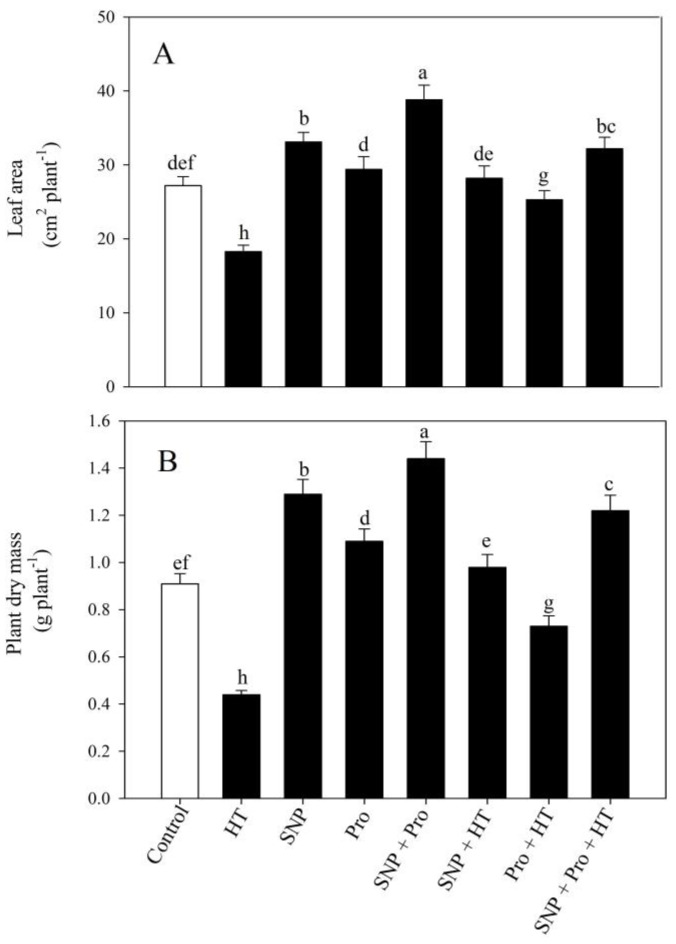
Leaf area (**A**) and plant dry mass (**B**) in wheat (*Triticum aestivum* L. var. WH 711) leaves treated with 50 µM SNP and/or 50 mM Pro in the presence (40 °C) or absence (28 °C) of heat stress at 30 DAS. Data are presented as treatment mean ± SE (n = 4). Data followed by same letter are not significantly different by LSD test at *p* < 0.05. SNP: sodium nitroprusside; Pro: proline; HT: heat stress; DAS: days after sowing.

In contrast to the control plants, exposure to high temperatures significantly hindered plant growth by reducing leaf area by 32.7% and dry mass by 51.6%. However, in both standard and high temperature circumstances, individual SNP and proline application improved plants’ leaf area and dry mass (Figure 1). Nevertheless, applying SNP and proline treatments during high temperature stress resulted in a substantial increase of 76% and 177% in leaf area and plant dry mass, respectively, as opposed to heat-exposed plants (Figure 1).

High temperature stress noticeably impacted the photosynthesis-related characteristics and chlorophyll content. High temperature stress lowered net photosynthesis (Pn) by 47.5%, stomatal conductance (Gs) by 28.5%, intercellular CO_2_ concentration (Ci) by 47.2%, SPAD value by 58.5%, Fv/Fm by 19.4%, and Rubisco activity by 36.7%, relative to control plants. Foliar application of SNP/proline to unstressed plants showed the greatest increase in the above-mentioned parameters compared to control plants. Furthermore, proportionate to the heat-stressed plants, plants exposed to both nitric oxide and high temperature showed an increase in Pn by 96.3%, Gs by 38.1%, Ci by 58.9%, SPAD by 71.3%, Fv/Fm by 22.4%, and in the activity of Rubisco by 63.3%. However, plants receiving Pro under high temperature stress improved the traits mentioned above by 68.7%, 25.9%, 67.6%, 50.1%, 15.6%, and 32.5%, respectively, relative to the only heat-treated plants. The administration of nitric oxide and proline showed enhancement in Pn by 156.1%, Gs by 62.6%, Ci by 193.1%, SPAD by 106.1%, Fv/Fm by 53.3%, and Rubisco activity by 92.6%, in contrast to the plants subjected to heat treatment (Table 1).

### 2.2. NO and Proline Mitigate the Oxidative Stress Connected with High Temperature Stress

**Figure 2:**
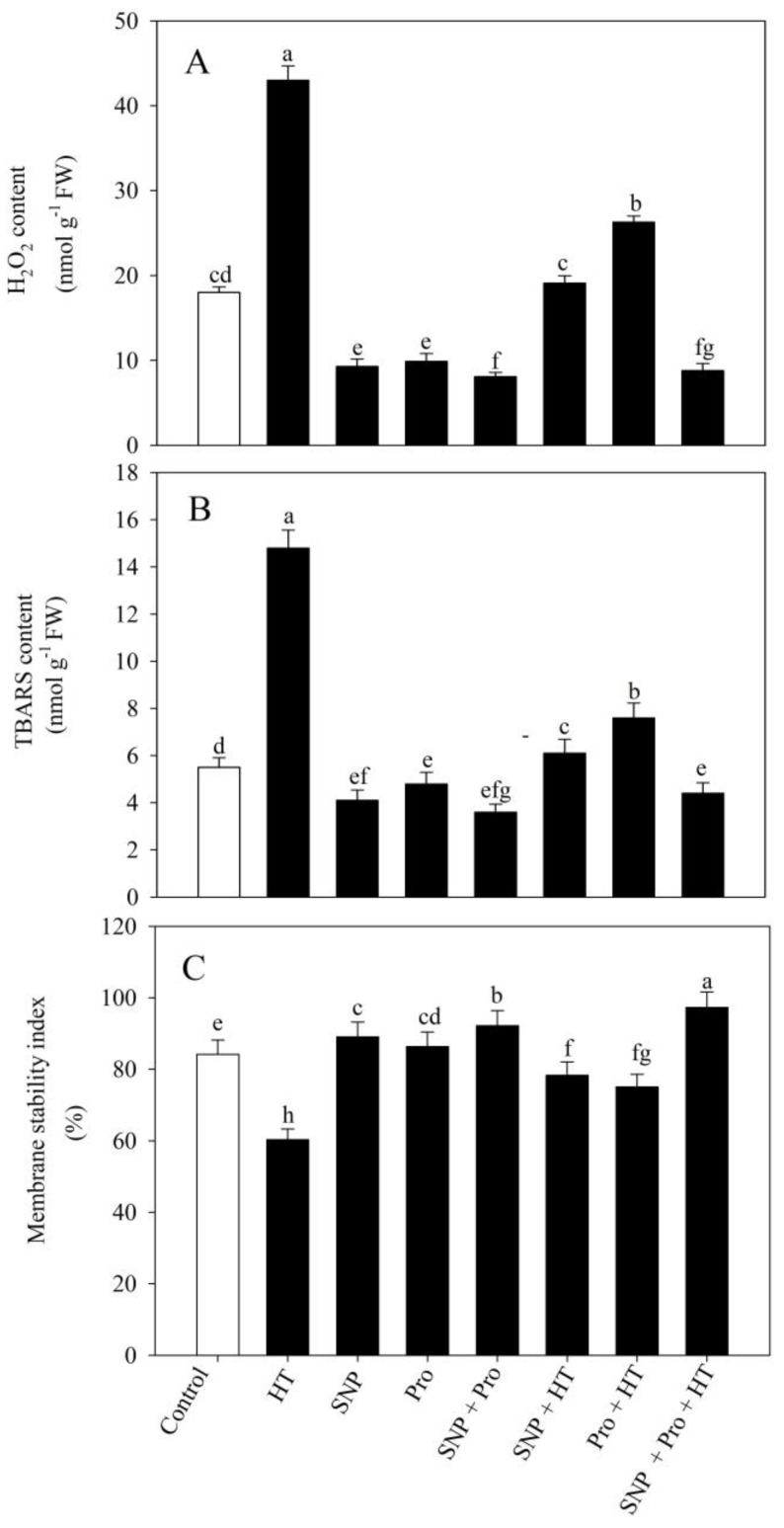
Content of H_2_O_2_ (**A**), TBARS (**B**) and membrane stability index (MSI) (**C**) in wheat (*Triticum aestivum* L. var. WH 711) leaves treated with 50 µM SNP and/or 50 mM Pro in the presence (40 °C) or absence (28 °C) of heat stress at 30 DAS. Data are presented as treatment mean ± SE (n = 4). Data followed by same letter are not significantly different by LSD test at *p* < 0.05. SNP: sodium nitroprusside; Pro: proline; HT: heat stress.

The content of hydrogen peroxide (H_2_O_2_) and thiobarbituric acid reactive substances (TBARS) was analyzed to quantify the degree of cellular destruction brought on by heat-induced oxidative stress. High temperature stress considerably enhanced H_2_O_2_ and TBARS content compared to the control plants by 138.8% and 169%, respectively. Individual treatment of SNP/proline under unstressed conditions significantly decreased the amount of H_2_O_2_ and TBARS by 48.3% and 45%, and 25.4% and 12.7%, respectively, compared to the control plant. The combined application of SNP and proline contributed to the lowest H_2_O_2_ and TBARS levels observed in unstressed plants, and that presented a 56% and 36% reduction in these parameters, respectively, compared to control plants. Foliar supply of SNP and proline individually to heat-stressed plants reduced this temperature-elicited oxidative stress by lowering H_2_O_2_ and TBARS relative to only heat-stressed plants, respectively, by 55.6% and 58.7%, and 38.8% and 48.6%. However, exogenous co-application of SNP and proline reduced this temperature-induced oxidative stress by showing a maximum reduction in H_2_O_2_ of 79.5% and TBARS of 72%, relative to heat-stressed plants (Figure 2).

### 2.3. Effect of NO and Proline on Membrane Stability Index (MSI) under Heat Stress

Compared to the control plants, the high temperature stress reduced the MSI by 29.4%. Individual applications of SNP and proline also raised MSI proportionate to the untreated plants by 4.0 and 2.0%, respectively. However, when NO was treated alongside proline, MSI increased by 7.0%, corresponding to the untreated plants.

Individual NO/proline supplementation on plants grown in heat-stressed conditions improved MSI by 29.8 and 24.7%, respectively, compared to only heat-stressed plants. Additionally, the combined application of SNP and proline supplementation under heat stress showed maximal improvement in MSI of 61.5% compared to plants grown under heat stress conditions (Figure 2).

### 2.4. NO and Proline Boost Activity of Antioxidant Enzymes under High Temperature Stress

**Figure 3:**
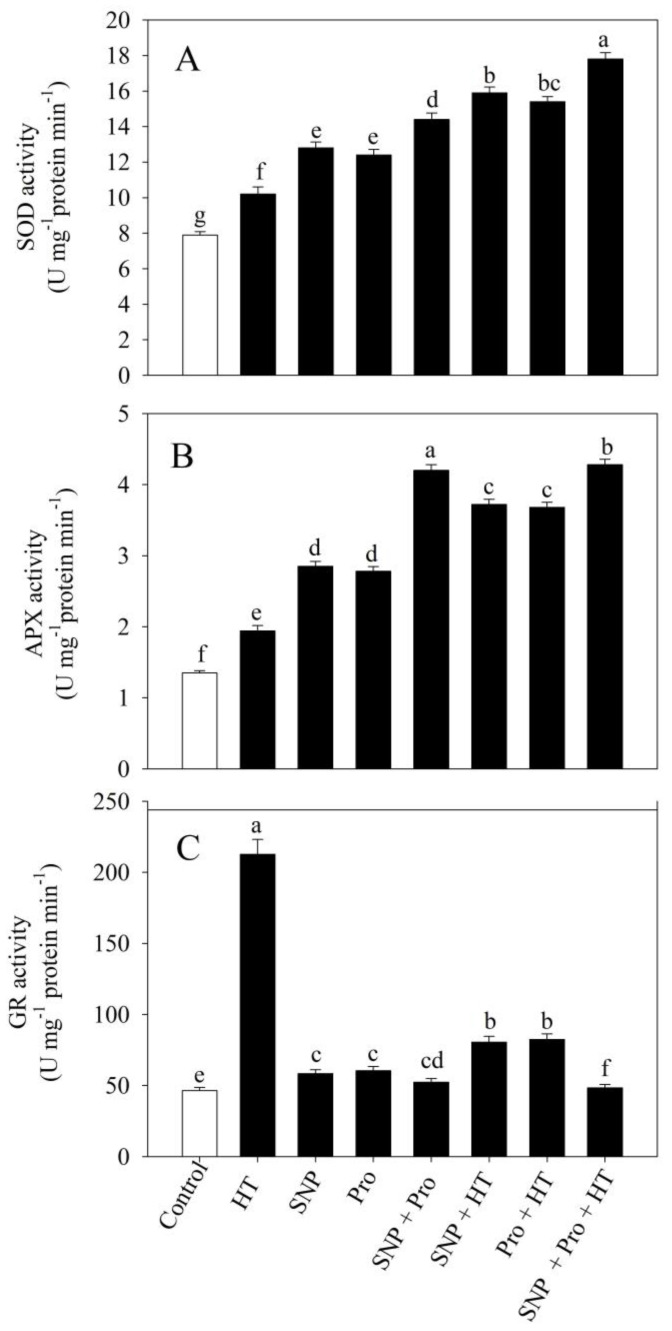
Activity of SOD (**A**), APX (**B**) and GR (**C**) in wheat (*Triticum aestivum* L. var. WH 711) leaves treated with 50 µM SNP and/or 50 mM Pro in the presence (40 °C) or absence (28 °C) of heat stress at 30 DAS. Data are presented as treatment mean ± SE (n = 4). Data followed by same letter are not significantly different by LSD test at *p* < 0.05. SOD: superoxide dismutase; APX: ascorbate peroxidase; GR: glutathione reductase; SNP: sodium nitroprusside; Pro: proline; HT: heat stress.

The activity of antioxidant enzymes was considerably enhanced against high temperatures with or without exogenous SNP and proline. The activity of superoxide dismutase (SOD), APX, and GR in plants increased under high temperature treatment by 30.7%, 43.7%, and 62.1% compared to the untreated plants, respectively (Figure 3).

The spray of SNP/proline singly on unstressed plants increased the activity of SOD by 62.2% and 57.1%, APX by 111.1% and 106%, and GR by 82.4% and 62.5, respectively, in contrast to control plants. The individual supplementation of SNP and proline on leaves of heat-stressed plants enhanced the activity of SOD by 55.8% and 50.9%, APX by 90.7% and 89.6%, and GR by 54.9% and 25% compared to heat-stressed plants. Furthermore, compared to plants exposed to high temperature stress, supplementing with SNP and proline enhanced the activity of antioxidant enzymes maximally by 74.5%, 120.6%, and 68.5%, respectively (Figure 3).

### 2.5. Influence of NO and Proline on NO Generation, ACS (Aminocyclopropane-1-Carboxylic Acid Synthase) Activity, and Ethylene Evolution under High Temperature Stress

**Figure 4:**
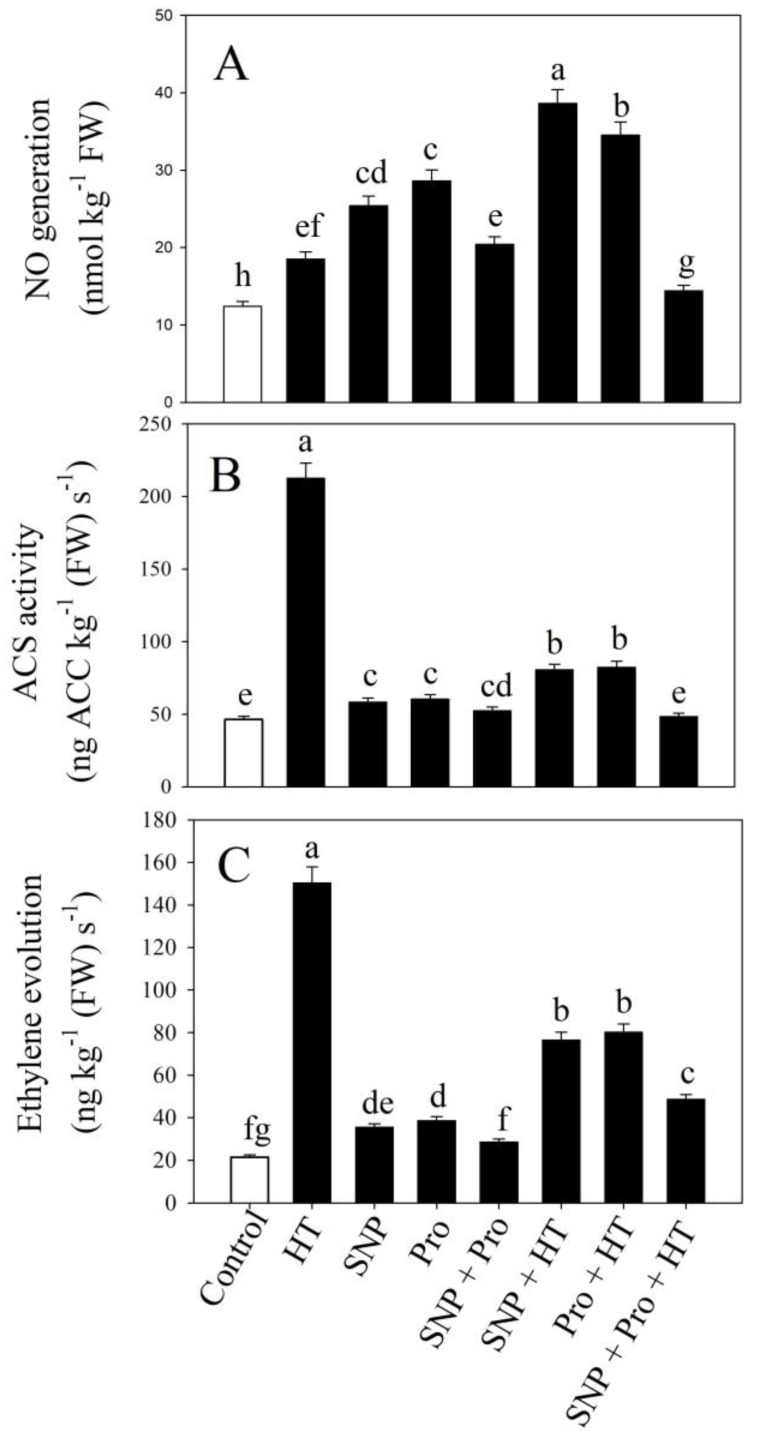
NO generation (**A**), ACS activity (**B**), and ethylene evolution (**C**) in wheat (*Triticum aestivum* L. var. WH 711) leaves treated with 50 µM SNP and/or 50 mM Pro in the presence (40 °C) or absence (28 °C) of heat stress at 30 DAS. Data are presented as treatment mean ± SE (n = 4). Data followed by same letter are not significantly different by LSD test at *p* < 0.05. NO: nitric oxide; ACS: aminocyclopropane-1-carboxylic acid synthase; SNP: sodium nitroprusside; Pro: proline; HT: heat stress.

Heat-stressed plants produced more NO, had more ACS activity, and evolved more ethylene than control plants. SNP and proline alone under unstressed conditions led to higher NO production, ACS activity, and ethylene evolution in contrast to control (Figure 4).

The individual SNP/proline supplementation in the presence of heat stress enhanced NO generation by 108% and 86.4%; however, the reduction was recorded in ACS activity by 62% and 61%, and ethylene evolution by 49% and 46.6%, respectively, in comparison to only heat-stressed plants. Moreover, plants treated with a combination of SNP and proline subjected to heat stress showed the greatest reductions in NO generation by 22.1%, ACS activity by 77.3%, and ethylene evolution by 67.6%, in comparison to plants subjected to heat stress (Figure 4).

### 2.6. NO and Proline Enhance Proline and Glycine Betaine (GB) Content under High Temperature Stress

**Figure 5:**
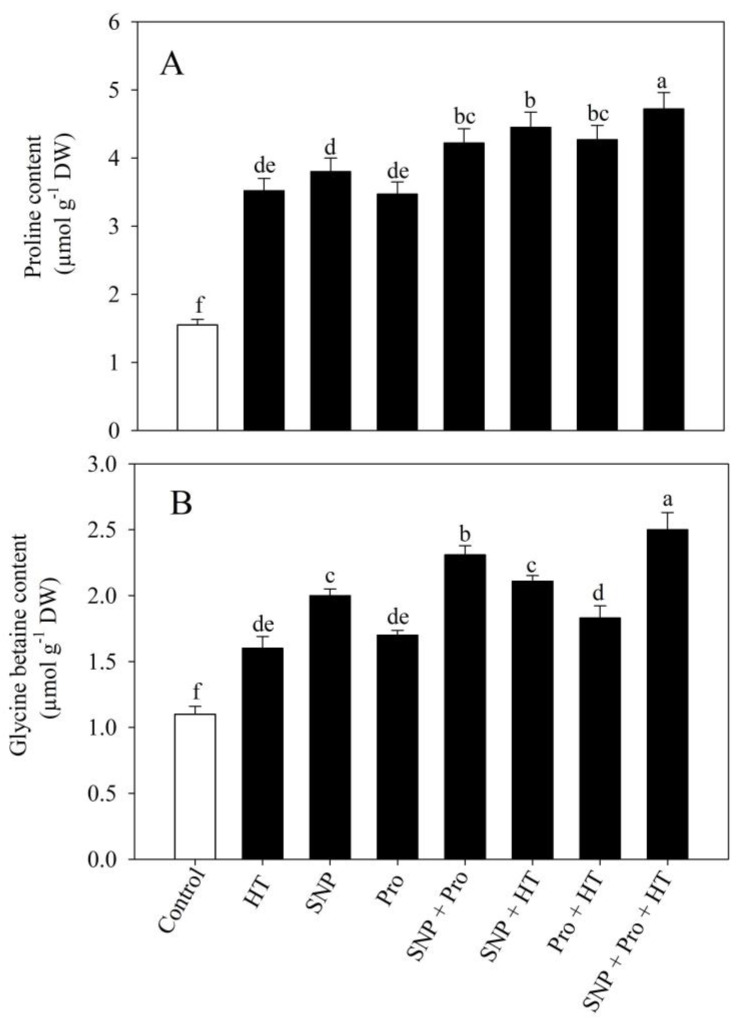
Content of proline (**A**) and glycine betaine (**B**) in wheat (*Triticum aestivum* L. var. WH 711) leaves treated with 50 µM SNP and/or 50 mM Pro in the presence (40 °C) or absence (28 °C) of heat stress at 30 DAS. Data are presented as treatment mean ± SE (n = 4). Data followed by same letter are not significantly different by LSD test at *p* < 0.05. SNP: sodium nitroprusside; Pro: proline; HT: heat stress.

The role of proline and GB content in preventing heat stress was examined. Proline content increased by 127.6% and GB by 45.4% compared to control plants. Proline content increased significantly after treatment with individual SNP and proline by 146.1% and 123.1%, and GB content increased by 82% and 55.1%, respectively, compared to the control. Under normal conditions, plants with exogenous SNP and proline displayed still more proline and GB than the control by 172.5% and 110%, respectively. Exogenous SNP/proline treatment to heat-stressed plants significantly increased proline and GB content compared to plants not subjected to heat stress. Treatment with SNP and proline under high temperature stress resulted in the highest significant increases in proline by 34.1% and GB by 56.1%, respectively, compared to only heat-treated plants (Figure 5).

### 2.7. Influence of NO and Proline on the Expression of GR Gene under Heat Stress

**Figure 6:**
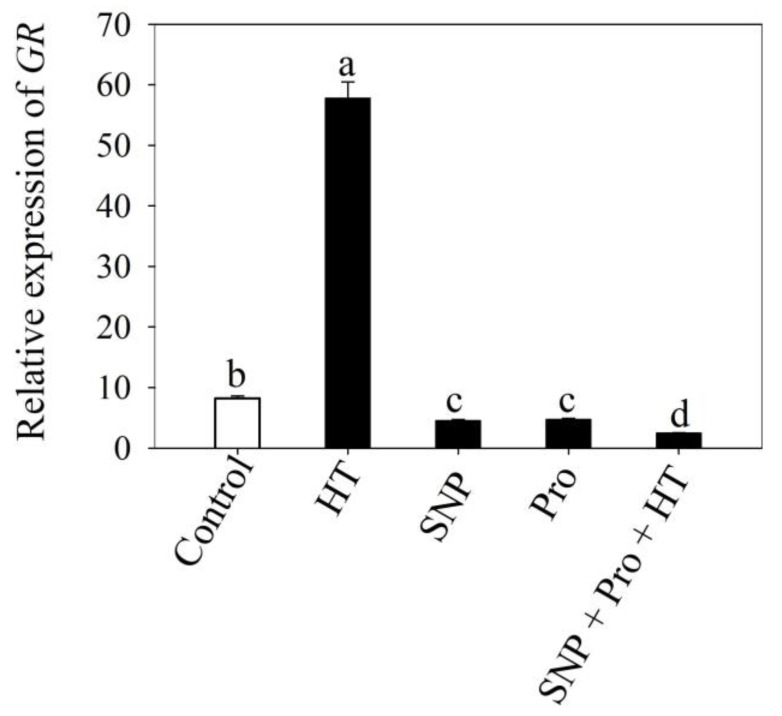
Relative expression of GR in wheat (*Triticum aestivum* L. var. WH 711) leaves treated with 50 µM SNP and/or 50 mM Pro in the presence (40 °C) or absence (28 °C) of heat stress at 30 DAS. Data are presented as treatment mean ± SE (n = 4). Data followed by same letter are not significantly different by LSD test at *p* < 0.05. GR: glutathione reductase; SNP: sodium nitroprusside; Pro: proline.

The impact on the relative expression of the GR gene by the external supply SNP with proline during heat stress was examined. Under heat stress, the expression of GR was highly upregulated in comparison to control plants. However, individual treatment of SNP/proline without heat stress showed a reduction in GR expression. Furthermore, co-application of SNP/proline under heat stress maximally reduced the GR expression compared to both unstressed and stressed plants (Figure 6).

### 2.8. Influence of NO and Proline on the Expression of Genes Encoding Core PSII Proteins

**Figure 7:**
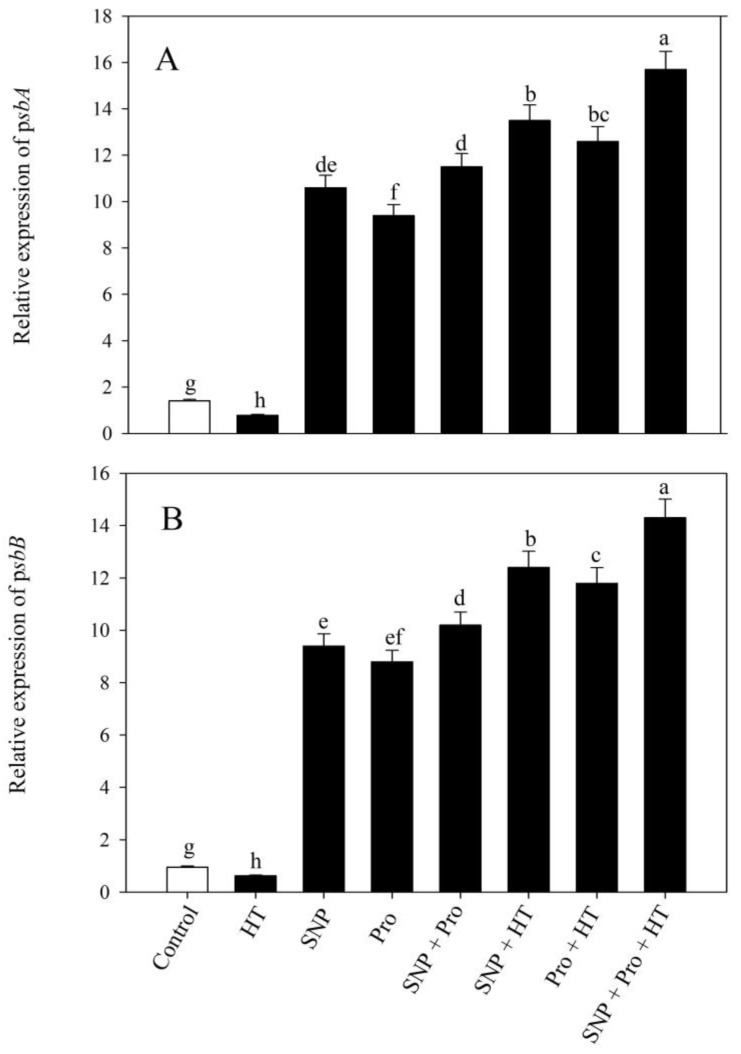
Relative expression of *psbA* (**A**) and *psbB* (**B**) in wheat (*Triticum aestivum* L. var. WH 711) leaves treated with 50 µM SNP and/or 50 mM Pro in the presence (40 °C) or absence (28 °C) of heat stress at 30 DAS. Data are presented as treatment mean ± SE (n = 4). Data followed by same letter are not significantly different by LSD test at *p* < 0.05. SNP: sodium nitroprusside; Pro: proline; HT: heat stress.

The exogenous administration of SNP and proline affects PSII activity and the expression of genes [6]. To better understand how SNP protects against high temperature stress, D1 and CP47 encoding genes *psbA* and *psbB* were examined for their expression in leaves. SNP supplementation substantially enhanced *psbA* and *psbB* expression compared to controls under normal circumstances; however, high temperature stress lowered the expression. Plants administered SNP under heat stress substantially increased the expression of *psbA* and *psbB*. Compared to all other treatments, plants subjected to the collective application of SNP and Pro under heat stress exhibited maximal enhancement in the expression levels of both genes (Figure 7).

### 2.9. Principal Component Analysis

**Figure 8:**
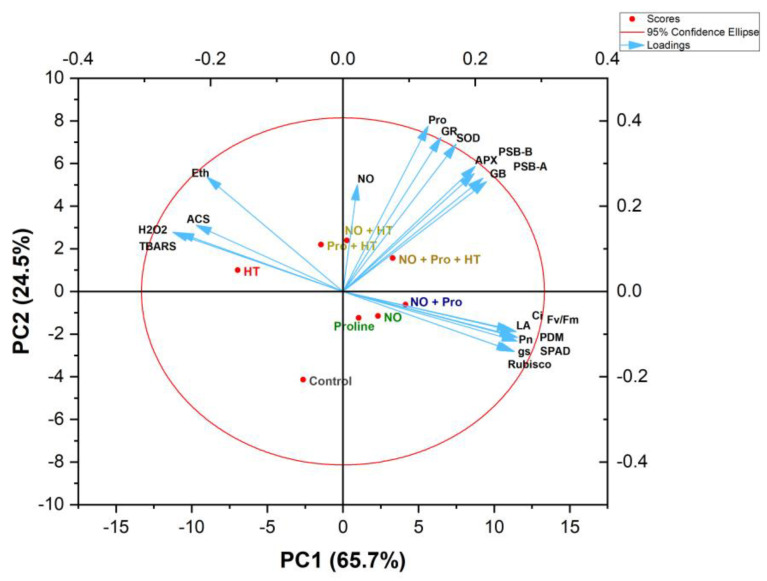
Biplots of principal component analysis (PCA) represent the relationship between different variables and treatments in wheat (*Triticum aestivum* L. cv. WH-711) treated with/without high temperature and 50 µM SNP and/or 50 mM proline. The variables included net photosynthesis (PN), stomatal conductance, intercellular CO_2_ concentration (C_int_), chlorophyll content, maximal PSII efficiency (Fv/Fm), Rubisco activity, plant dry mass, leaf area, H_2_O_2_ content, thiobarbituric acid reactive substances (TBARS) content, membrane stability index, superoxide dismutase (SOD), ascorbate peroxidase (APX), glutathione reductase (GR), nitric oxide (NO) generation, aminocyclopropane-1-carboxylic acid synthase (ACS) activity, ethylene evolution, proline (Pro) content, glycine betaine (GB) content, relative expression of GR, *psbA*, *psbB*.

A PCA was conducted to analyze the variability of collected information and the interrelation between the different treatments and attributes). The two components (PC1 and PC2) accounted for 90.2% of the total data variability induced by different treatments (Figure 8). PC1 encompassed 65.7% of the variation, while PC2 accounted for 24.5% of the total variation. The biplot was divided into three clusters. The oxidative stress indicators such as H_2_O_2_, TBARS and ethylene content, and ACS activity were clustered together, and they were proximate to heat stress treatment. On the other hand, leaf area and photosynthetic attributes were clustered together, and antioxidants were placed amid oxidative stress parameters and growth and photosynthetic parameters. The PCA plot indicated a favorable correlation among the parameters of plant growth, photosynthesis, chlorophyll fluorescence; the activity of GR, SOD and APX; expression level of *psbA* and *psbB*; and the content of NO, Pro, and GB (Figure 8). Plant growth and photosynthetic attributes, along with oxidative stress parameters, showed an unfavorable correlation with each other, and antioxidants were positioned in amidst them, indicating their function in assuaging HT stress. Furthermore, intrinsic NO showed a positive correlation with enzymatic and non-enzymatic antioxidants. Overall, GB and Pro showed a close association with endogenous NO in plants under heat stress. Amongst the treatments, HT was linked to oxidative stress and ethylene buildup; however, NO + Pro + HT exhibited a robust association with antioxidants, implying the effectiveness of the NO + Pro + HT treatment in mitigating HT stress (Figure 8). On the other hand, NO and Pro treatments were close to growth and photosynthetic parameters.

### 2.10. Pearson Correlation

**Figure 9:**
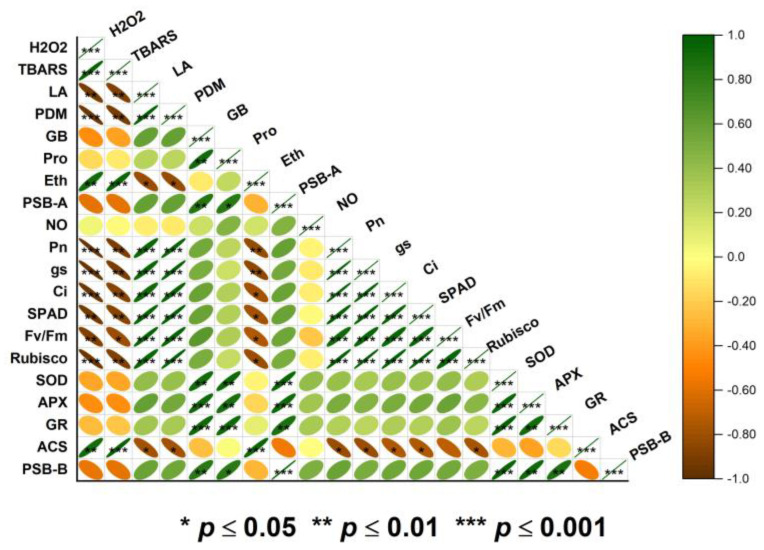
Pearson correlation matrix represents the relationship among different variables and treatments in wheat (*Triticum aestivum* L. cv. WH-711) treated with/without high temperature and 50 µM SNP and/or 50 mM proline. The variables included net photosynthesis (PN), stomatal conductance, intercellular CO_2_ concentration (C_int_), chlorophyll content, maximal PSII efficiency (Fv/Fm), Rubisco activity, plant dry mass, leaf area, H_2_O_2_ content, thiobarbituric acid reactive substances (TBARS) content, membrane stability index, superoxide dismutase (SOD), ascorbate peroxidase (APX), glutathione reductase (GR), nitric oxide (NO) generation, aminocyclopropane-1-carboxylic acid synthase (ACS) activity, ethylene evolution, proline (Pro) content, glycine betaine(GB) content, relative expression of GR, *psbA*, *psbB*.

A heat map using Pearson correlation was generated to study the correlation between growth, photosynthetic, oxidative stress, and antioxidant parameters with heat stress (Figure 9). The results indicated that HT stress had a strong relationship with oxidative stress attributes such as H_2_O_2_ and TBARS content (Figure 9). In contrast, the oxidative stress markers (H_2_O_2_, and TBARS content) were found to have a negative correlation (*p* ≥ 0.05, *p* ≥ 0.01, and *p* ≥ 0.001) with several attributes including leaf area, Pn, Ci, gs, chlorophyll content, Rubisco activity, and Fv/Fm. Meanwhile, the antioxidants (SOD, APX, GR, and GSH) demonstrated a significant (*p* ≥ 0.05, *p* ≥ 0.01, and *p* ≥ 0.001) favorable relationship with the plant’s overall growth and photosynthetic features (Figure 9). Endogenous NO showed a strong correlation with the expression of genes of PSII (PSB-A and PSB-B). Moreover, endogenous NO also indicated a positive correlation with GB and Pro content, suggesting their potential involvement in thermo-tolerance.

## 3. Discussion

According to the IPCC (Intergovernmental Panel on Climate Change) report, global warming is expected to exceed by 1.5–2.0 °C during the 21st century if greenhouse gas emission is not controlled [25]. With the increase in temperature, a notable decrease in photosynthesis and growth is expected, which ultimately hinders crop yield [3,4,6]. Wheat is considered a staple food, which is sensitive to heat stress. It has been shown that an increase of 1 °C in the global mean temperature will result in 8.0% and 3.0% decreases in wheat yield alone in India and China, respectively [23,26]. Thus, temperature stress is detrimental to wheat cultivation, and initiatives should be taken and prioritized to mitigate its adverse effects.

Heat stress results in an overproduction of ROS, which damages proteins, DNA, and cells by oxidizing lipids, harming plant photosynthesis and growth [27,28]. They also serve as a signaling component to activate the antioxidant system, enabling plants to withstand and respond to abiotic stress challenges [3]. Further research into the processes that could improve antioxidative metabolism is necessary because the potency of these antioxidants is insufficient to diminish oxidative stress. Osmolytes, in addition, are a crucial element that preserves the cell’s redox state by serving as an antioxidant and maintaining osmotic balance. Plant hormones may also aid in lowering ROS levels by inducing antioxidant defense and osmolytes accumulation signals.

Amongst various signaling molecules, NO is receiving increased attention owing to its efficacy as a stress reliever [29]. However, the effectiveness of NO as a possible signaling molecule depends on its concentration [29]. The present study examined the impact of nitric oxide and proline separately and together in reducing heat stress. However, little is known about how exogenous supplementation with nitric oxide and proline affects antioxidant metabolism and photosynthesis in heat stress. We analyzed from the current study that proline’s action was reliant on nitric oxide, and that both nitric oxide and proline reduced heat stress impacts.

In this study, we found that heat stress reduces photosynthesis and growth of wheat plants. Similar effects were observed in previous studies, which was due to the adverse impact of heat on thylakoid structures, protein complexes of chloroplasts, PS II, and Rubisco activity [3,7,29,30]. To combat heat stress, the plant’s photosynthetic efficiency needs to be increased. In the current study, we found that NO and proline application improved photosynthesis by protecting Rubisco activity, chlorophyll content and PSII efficiency, and upregulating the expression of PSII proteins. Similar results were noted by Sehar et al. [6] and Gautam et al. [7]. They observed that NO and proline prevent chlorophyll loss and protect PSII activity and expression, increasing photosynthesis and quantum yield. In addition, this study observed that NO and proline application accumulate more osmolytes such as proline and glycine betaine. The findings of this report align with the results obtained by Iqbal et al. [4]. Iqbal et al. [4] showed that NO supplementation alters osmoprotectants and the antioxidant defense machinery, which controls the metabolism of ROS and RNS, and gives plants the ability to withstand heat stress. Although the involvement of NO and proline in these processes under extreme heat is not well-defined, it is crucial for the integrity and successful operation of the photosynthetic system that chlorophyll breakdown and biosynthesis are balanced. Thus, it may be said that the combination of NO and proline enhances photosynthesis under heat stress, which improves plant growth and alleviates heat stress’s detrimental results.

Heat stress enhances NO generation; furthermore, the application of SNP and proline individually under heat stress shows a maximum increase in NO generation. According to published research, exposure to high temperatures causes an increase in NO buildup in a variety of plant species such as rice and wheat [3,4,12]. Although the research emphasizes the release of heat-activated NO as a crucial response for enhancing plant stress tolerance [12,31], a detailed study is lacking. According to a report, NO levels are elevated with heat stress, and the induction of heat shock protein (HSP) through exogenous H_2_S is dependent on NO [32]. However, in our experiment, the co-application of SNP and proline under heat stress reduced the NO generation. This implies that a combination of these may reduce the repercussions of heat by reducing oxidative stress and maintaining osmotic homeostasis, which reduces NO generation. Moreover, the present study observed that heat stress enhances ACS enzyme activity and excess ethylene evolution, referred to as stress ethylene. The production of stress ethylene hindered plant growth and plant physiology [3]. Previous studies have demonstrated the significance of both optimal levels of ethylene and ethylene generation induced by stress [3,6,33]. The addition of SNP/proline singly decreases the ethylene level. However, the combined application of SNP and proline reduces ACS activity, and thus brings down ethylene to the optimal level. The optimum level of ethylene aids to reduce heat-induced oxidative stress and confers thermotolerance. This result is in agreement with the previous report of Gautam et al. [3] and Sehar et al. [6].

In the examined wheat plant, high temperature stress was found to augment the oxidative stress as seen by elevated H_2_O_2_ and TBARS levels; nevertheless, the same values were decreased in plants treated with NO. However, this reaction was not adequate to reduce the oxidative stress brought on by the high temperatures. Oxidative stress, which disturbs the redox equilibrium of cells, is brought on by the increased production of ROS under heat stress, including the superoxide anion radical (O^2−^) and hydrogen peroxide (H_2_O_2_) [34]. These free radicals cause harm to membranes and macromolecules, which has negative impacts on plant metabolism and yield [35,36]. As a result, the ability of antioxidant enzymes or molecules to remove ROS more quickly typically determines whether a plant will survive in a heat-stressed environment. Heat stress is just one of many stress responses where NO’s antioxidant role has been well-documented [3,4,7,35,37,38,39]. Due to its capacity to neutralize the deleterious impact of ROS, NO has appeared to play two important roles in reducing the effects of heat-induced oxidative stress [40]. The first role is the preservation of cellular redox homeostasis, and the second is NO-promoted protection from oxidative stress by stabilizing the carotenoids during heat stress [40] Carotenoids are recognized as agents that prevent photo-oxidative damage because of their capacity to scavenge free radicals [40]. Therefore, higher amounts of carotenoids provide superior heat stress defense. In *Chrysanthemum morifolium* under heat stress, foliar application of SNP was found to increase carotenoid concentrations, resulting in improved heat tolerance [41]. Plants exposed to high temperature stress showed increased activity of antioxidant enzymes such SOD, APX, and GR. Iqbal et al. [4] manifested that NO supplementation significantly mediated heat stress tolerance by regulating the accumulation of osmolytes, and upregulating the activity and gene expression of antioxidant enzymes. This led to the increased photosynthetic performance of plants under heat exposure. The current study’s findings validated that the NO signaling system can modulate ROS scavenger enzymes, enabling wheat seedlings to adapt to heat-induced oxidative stress. The results also showed that exogenous treatment of NO and proline individually and in combination under heat stress elevated SOD, APX, and GR activity and their gene expression. Although the information that was available indicated that sustaining cellular NO levels was a crucial part of plants’ responses to heat stress, further research is required to determine how precisely ROS/NO are regulated when exogenous NO donors are used.

We ran our data for principal component analysis to further confirm the role of NO and proline in heat stress tolerance. The relation between all paired attributes, such as antioxidant metabolism, and their expression positively responded to photosynthetic and growth metrics. In contrast, it was negatively correlated with oxidative stress biomarkers. The results obtained hold considerable importance for wheat cultivation in areas experiencing heat stress by adopting the inputs of the tested biostimulants. The findings could potentially lead to further efforts to develop a more efficient system for managing crop stress in the future.

## 4. Material and Methods

### 4.1. Plant Material, Growth Conditions, and Treatments

Wheat (*Triticum aestivum* L. cv. WH-711) was sourced from the National Seeds Corporation, New Delhi, India. Prior to sowing, the seeds were disinfected using 0.01% mercuric chloride and washed with deionized water. Then, seeds were planted in earthen pots containing acid-rinsed sand, and were kept at the experimental area of the Department of Botany, Aligarh Muslim University, Aligarh, India, with day/night temperatures of 25/18 ± 3 °C, 12 h photoperiod (680 µmol m^−2^ s^−1^), and relative humidity of 65 ± 5%. Two plants were grown per pot, and they were irrigated with 300 mL of full-strength Hoagland’s solution on alternate days.

In this experiment, the plants were exposed to heat stress at 10 DAS (days after sowing) by maintaining a temperature of 40 °C for six hours regularly for 15 days (with the rest of the growth conditions remaining the same), followed by five days of growth at their optimal temperature of 28 °C. Throughout the experimental period (i.e., 30 days), control plants were kept at a constant temperature of 28 °C under the above-mentioned growth conditions. At 20 DAS, the plants were treated with 50 µM nitric oxide (NO) and 50 mM proline (Pro), either alone or in combination on the foliage of plants exposed to heat stress and unstressed conditions. The concentrations of 50 µM NO and 50 mM proline were based on our previous findings [6,18]. To ensure better hormone absorption, a surfactant Teepol was added to all treatments at a concentration of 0.5%. A completely randomized block design was utilized in performing the experiment, with each treatment having four replicates (n = 4).

### 4.2. Measurement of Photosynthetic and Growth Indices

The photosynthetic attributes (net photosynthetic rate, stomatal conductance, and intercellular CO_2_ concentration) were measured in the uppermost fully expanded leaf using Infra Red Gas Analyzer (CID-340, Photosynthesis System, Bio-science, Washington, WI, USA). The concentration of atmospheric CO_2_ was 380 µmol mol^−1^ at the time of the observations (between 11:00 and 12:00 h), with 70% relative humidity, 782 µmol m^−2^ s^−1^ PAR and 28 °C temperature.

The maximum PS II efficiency was recorded through a chlorophyll fluorometer (Junior-PAM, Heinz Walz, Germany) and expressed as Fv/Fm. The methodology used is outlined in Iqbal et al. [4].

The activity of Rubisco (EC 4.1.1.39) was estimated using a spectrophotometric approach described by Usuda [42]. The assay of the enzyme was carried out by observing the oxidation of NADH at 30 °C at 340 nm after the addition of enzyme extract and 0.2 mM ribulose-1,5-bisphosphate (RuBP). The details are given in Iqbal et al. [4].

The plants were carefully uprooted and rinsed to remove all debris to assess the growth. The samples were then dehydrated at 80 °C in the oven until they reached a stable weight to quantify the dry biomass of plants. Leaf area was determined by leaf area meter (LA211, Systronics, New Delhi, India).

### 4.3. Determination of Proline and Glycine Betaine Content

The ninhydrin technique was used to determine the proline content by Bates et al. [43]. Fresh leaf tissue weighing 300 mg was homogenized in 3.0 mL of 3% sulphosalicylic acid and then centrifuged for 12 min at 11,500× *g*. The resulting supernatant filtrate was mixed with 1.0 mL each of acid ninhydrin and glacial acetic acid in a test tube, and incubated for 1 h in a water bath at 100 °C. Afterward, for the extraction of the mixture, 4.0 mL of toluene was added and vigorously stirred and left for 5–10 min. L-proline was used as a reference to measure the absorbance at 520 nm on a spectrophotometer.

Utilizing Grieve and Grattan’s [44] approach, glycine betaine (GB) was quantified by developing the complex of the betaine–peridotite. A test tube containing 20 mL of deionized water and 0.5 g of oven-dried leaves was mechanically agitated for 24 h at room temperature and then filtered. To dilute the filtrates, they were treated with 1:12 N H_2_SO_4_. A 0.5 mL portion was added to centrifuge tubes and chilled in ice water for 1 h. After adding 0.2 mL of cold KI-I_2_ reagent, the reactants were gently stirred, and the tubes were kept at 4 °C for 16 h before being centrifuged at 10,000× *g* for 15 min at 0 °C. The supernatant was cautiously aspirated, and after 2 h, the absorbance at 365 nm was measured. GB reference standards (50–200 g mL^−1^) were prepared in 2 N H_2_SO_4_.

### 4.4. Quantification of Hydrogen Peroxide Content, Lipid Peroxidation and Membrane Stability Index

The H_2_O_2_ quantity was calculated by applying the methodology of Okuda et al. [45], the particulars of which are stated in Sehar et al. [6]. According to Dhindsa et al.’s [46] method, the amount of TBARS was calculated to measure the degree of peroxidation of lipids. Supplementary File S1 contains the method’s specifics.

The approach of Das and Uprety [47] was used to calculate the membrane stability index. Small, round discs of freshly cut leaves were created. After incubating samples at 40 °C, their electrical conductivity (C1) was measured. Subsequently, the samples were transferred to a water bath set at a temperature of 100 °C for 15 min, and their electrical conductivity (C2) was recorded again. The MSI (membrane stability index) was estimated by applying the given formula.

MSI = [1 − (C1/C2)] × 100

### 4.5. Assay of Antioxidants Enzymes Activity

Fresh leaves were homogenized with an extraction buffer comprising 0.05% (*v*/*v*) Triton X-100 and 1% (*w*/*v*) PVP in potassium-phosphate buffer (100 mM, pH 7.0) using a cold mortar and pestle. After centrifugation, the supernatant was used for the SOD and GR (EC; 1.6.4.2) enzymes assay. Extraction buffer was used in addition to 2.0 mM ascorbate for the measurement of APX (EC; 1.11.1.11). The activity of SOD was assayed by the method of Beyer and Fridovich [48] and Giannopolitis and Ries [49]. The activity of APX was determined following the method of Nakano and Asada [50], by recording the decrease in the absorbance of ascorbate at 290 nm. The activity of GR was determined by the method of Foyer and Halliwell [51] by monitoring the glutathione-dependent oxidation of NADPH at 340 nm. The procedure is mentioned in full in Supplementary File S1.

### 4.6. Determination of NO (Nitric Oxide) Generation, ACS (Aminocyclopropane-1-Carboxylic Acid Synthase) Activity, and Ethylene Evolution

The Zhou et al. [52] method was slightly adjusted to evaluate NO content by measuring nitrite concentration. Healthy leaves were pulverized to powder and then dissolved in 3.0 mL of chilled, 50 mM zinc acetate (4%), acetic acid buffer (pH 3.6) solution. Following centrifugation at 11,500× *g* at 4 °C for 15 min, the supernatant was removed, and the particulates were washed with extraction buffer (1.0 mL) and centrifuged once more. By mixing in 0.1 g of charcoal, the supernatants from the two spun were neutralized and the resulting filtrate was extracted following a short vortex. Greiss reagent was added to 1.0 mL of filtrate and kept at room temperature for 30 min. Before centrifuging the sample again, the absorbance at 540 nm (1.0 mL) was recorded in order to calculate the NO concentration using a standard curve of sodium nitrite.

ACS (EC, 4.4.1.14) was assessed in accordance with the technique of Avni et al. [53] and Woeste et al. [54]. Leaf tissue (5.0 g) was homogenized in 100 mM HEPES buffer (pH 8.0) containing 4 mM DTT, 2.5 mM pyridoxal phosphate, and 25% PVP. The homogenized material was centrifuged at 12,000× *g* for 15 min. Then, 1 mL of the supernatant was placed in a 30 mL tube and 0.1 mL of 5 mM S- adenosyl methionine (Ado-Met) was added and incubated for 2 h at 22 °C. The ACC formed was determined by its conversion to ethylene by the addition of 0.1 mL of 20 mM HgCl_2_ followed by the addition of 0.1 mL of a 1:1 ratio of saturated NaOH/NaCl and placed in an ice bath for 10 min. In the control set, Ado-Met was not added.

To measure ethylene, the procedure given in Fatma et al., [55] was followed. The 0.5 g of cut leaf material was placed into 30 mL tubes containing moist paper to reduce tissue evaporation. The tubes were stoppered with rubber caps and exposed to light for 2 h under the same growth conditions as the plants. A 1 mL gas sample was then extracted from the tubes using a hypodermic syringe and analyzed using a Nucon 5700 gas chromatograph (Nucon Engineers, New Delhi, India) equipped with a 1.8 m Porapack ^TM^ N (80–100 mesh) column (Sigma-Aldrich, Saint Louis, MO, USA), a flame ionization detector, and a data station. Nitrogen was used as the carrier gas, with flow rates of 30, 30, and 300 mL min^−1^ for nitrogen, hydrogen, and oxygen, respectively. The detector was calibrated to 150 °C. Ethylene was detected based on its retention time and quantified by comparing peaks from the standard ethylene concentration.

### 4.7. RNA Isolation and cDNA Synthesis

TRIzol reagent from Ambion, Life Technologies was used for total RNA extraction from leaves following the manufacturer’s instructions. The quantity of RNA was determined using a Nanodrop spectrophotometer from Thermo Scientific, Waltham, MA, USA. The first strand cDNA was generated from 1 μg of total RNA of both control and treated samples, and the template was synthesized using a reaction mixture containing 20 U/μL Moloney Murine Leukemia virus reverse transcriptase (MuMLV) enzyme from Fermentas, Maryland, USA, at 42 °C for 50 min, followed by 70 °C for 10 min. The reverse transcriptase was performed with 2.5 μM Oligo (dT) 18 primer (Fermentas, USA) and 10 mM dNTPs. Primers for gene expression analysis were designed using IDT online primer design software, and the cDNA sequences of selected genes were obtained from NCBI.

### 4.8. Quantitative Real-Time PCR Analysis

Real-time PCR (RT-PCR) was performed in a 96-well reaction plate (Roche, Mannheim, Germany) on a thermal cycler (Light cycler 480 II, Roche, Germany). The setup consisted of reaction a mixture (20 µL) of ×10 reaction buffer, 10 µL cDNA template, 1 mM MgCl_2_, 2 mM dNTPs, 1 µL Sybr green (×10) (Thermo Fisher Scientific, Waltham, MA, USA) 0.35 µM each of forward and reverse primers and 5 U Taq polymerase (Bio-rad, Hercules, CA, USA). To normalize the quantification, the actin DNA fragment amplified by β-actin forward and β-actin reverse primers was used as an internal control for all genes. The PCR cycling conditions were as follows: denaturation at 95 °C for 3 min, 40 cycles of 95 °C (20 s), 66 °C (1 min), and 72 °C (1 min), and a final extension at 72 °C for 5 min. The amplified product was analyzed on a 1.2% agarose gel, and the specificity of the amplicons was verified by melting curve analysis (60 to 95 °C) after 40 cycles. The RT-PCR reactions were performed in triplicate, using gene-specific primers and actin primers as an inter-control. The data were analyzed by comparing the expression of the gene of interest in in the treated sample with that in the untreated control, with normalization of the internal control (actin). The sequence of primer pairs used for quantitative RT-PCR is given in Supplementary File S1.

### 4.9. Statistical Analysis

Data were subjected to statistical analysis utilizing analysis of variance (ANOVA) by means of SPSS 17.0 for Windows, and presented as the mean value along with the standard error (mean ± SE), with a sample size of n = 4. The least significant difference (LSD) was calculated for the significant data at *p* < 0.05. Bars which share the same character are not considered significantly different by the LSD test at *p* < 0.05. Principal component analysis between different variables was performed using Origin Pro (v 9.8) for Windows.

## 5. Conclusions

In conclusion, the present outcomes showed that NO with proline enhanced photosynthetic and growth responses in normal and heat-stressed plants. Plants experienced oxidative stress due to heat-mediated increased ROS production. Exogenous NO supplementation considerably reduced heat-induced detrimental effects on photosynthesis and plant growth, and successfully reduced the repercussions of heat stress when paired with proline. Higher expression of *psbA* and *psbB* was one way that NO and proline’s stimulating effects on PSII photochemistry were seen. Through their combined effects on antioxidant metabolism, NO production, ACS activity, and ethylene synthesis, NO plus proline supplementation were most helpful in improving heat stress tolerance. This work offers a method for treating heat stress in wheat plants, and elaborates on the relationship between proline and the metabolism of antioxidants, which may be utilized to alter the plant genotype to make it more tolerant to heat stress.

## Figures and Tables

**Table 1:** Net photosynthesis, stomatal conductance, intercellular CO_2_ concentration, chlorophyll content, maximum efficiency of PSII and ribulose 1, 5 bisphosphate carboxylase/oxygenase (Rubisco) activity of wheat (*Triticum aestivum* L. var. WH 711) leaves treated with 50 µM SNP and/or 50 mM Pro in the presence (40 °C) or absence (28 °C) of heat stress at 30 DAS. Data followed by same letter are not significantly different by LSD test at *p* < 0.05. SNP: sodium nitroprusside; Pro: proline; HT: heat stress; DAS: days after sowing.

Treatments	Net Photosynthesis (µmol CO_2_ m^−2^ s^−1^)	Stomatal Conductance (mmol m^−2^ s^−1^)	Intercellular CO_2_ Concentration (µmol mol^−1^)	Chlorophyll Content (SPAD Value)	Maximum Quantum Yield Efficiency of PSII (Fv/Fm)	Rubisco Activity (µmol CO_2_ mg^−1^ Protein min^−1^)
Control	12.2 ± 0.78 ^d^	358 ± 16.1 ^d^	216 ± 10.7 ^d^	29.8 ± 1.6 ^d^	0.72 ± 0.040 ^d^	39.2 ± 2.1 ^d^
HT	6.4 ± 0.64 ^g^	256 ± 14.3 ^g^	114 ± 6.47 ^g^	18.8 ± 1.4 ^g^	0.58 ± 0.030 ^g^	24.8 ± 1.6 ^g^
SNP	17.2 ± 1.09 ^b^	436 ± 18.9 ^b^	352 ± 16.9 ^b^	43.8 ± 2.8 ^b^	0.86 ± 0.050 ^b^	54.4 ± 2.6 ^b^
Pro	16.5 ± 0.84 ^c^	421 ± 17.8 ^c^	338 ± 14.8 ^c^	39.6 ± 2.1 ^c^	0.78 ± 0.480 ^c^	48.8 ± 2.4 ^c^
SNP + Pro	18.2 ± 0.99 ^a^	464 ± 20.6 ^a^	378 ± 17.19 ^a^	48.8 ± 3.4 ^a^	0.98 ± 0.060 ^a^	56.8 ± 2.8 ^a^
SNP + HT	12.6 ± 0.87 ^d^	354 ± 16.8 ^d^	232 ± 11.7 ^d^	32.2 ± 1.8 ^d^	0.71 ± 0.041 ^d^	40.4 ± 2.3 ^d^
Pro + HT	10.8 ± 0.86 ^e^	322 ± 15.2 ^e^	191 ± 10.8 ^e^	28.2 ± 2.1 ^e^	0.67 ± 0.036 ^e^	32.9 ± 2.2 ^e^
SNP + Pro + HT	16.4 ± 0.94 ^c^	416 ± 18.2 ^c^	334 ± 13.3 ^c^	38.8 ± 1.9 ^c^	0.89 ± 0.050 ^c^	47.8 ± 2.4 ^c^

## Data Availability

Data are contained within the article and Supplementary Material.

## Supplementary Materials

Page: 17 The following supporting information can be downloaded at: https://www.mdpi.com/article/10.3390/plants12061256/s1, Supplementary File S1: The detailed methodology and Table S1. Primer pairs used for quantitative RT-PCR. Ref. [56] cite in Supplementary Materials.

## References

[1] de Almeida L.M.M., Coquemont-Guyot M., Elie N., Morvan-Bertrand A., Avice J.C., Mollier A., Brunel-Muguet S. (2023). Repeated heat stress events during the reproductive phase impact the dynamic development of seeds in *Brassica napus* L. Plant Sci..

[2] Fatma M., Iqbal N., Sehar Z., Alyemeni M.N., Kaushik P., Khan N.A., Ahmad P. (2021). Methyl jasmonate protects the PS II system by maintaining the stability of chloroplast D1 protein and accelerating enzymatic antioxidants in heat-stressed wheat plants. Antioxidants.

[3] Gautam H., Fatma M., Sehar Z., Mir I.R., Khan N.A. (2022). Hydrogen sulfide, ethylene, and nitric oxide regulate redox ho-meostasis and protect photosynthetic metabolism under high temperature stress in rice plants. Antioxidants.

[4] Iqbal N., Sehar Z., Fatma M., Umar S., Sofo A., Khan N.A. (2022). Nitric Oxide and Abscisic Acid Mediate Heat Stress Tolerance through Regulation of Osmolytes and Antioxidants to Protect Photosynthesis and Growth in Wheat Plants. Antioxidants.

[5] Sehar Z., Gautam H., Iqbal N., Alvi A.F., Jahan B., Fatma M., Albaqami M., Khan N.A. (2022). The functional interplay between ethylene, hydrogen sulfide, and sulfur in plant heat stress tolerance. Biomolecules.

[6] Sehar Z., Gautam H., Masood A., Khan N.A. (2022). Ethylene and proline-dependent regulation of antioxidant enzymes to mitigate heat stress and boost photosynthetic efficacy in wheat plants. J. Plant Growth Regul..

[7] Gautam H., Sehar Z., Rehman M.T., Hussain A., AlAjmi M.F., Khan N.A. (2021). Nitric oxide enhances photosynthetic nitrogen and sulfur-use efficiency and activity of ascorbate-glutathione cycle to reduce high temperature stress-induced oxidative stress in rice (*Oryza sativa* L.) plants. Biomolecules.

[8] Jegadeesan S., Chaturvedi P., Ghatak A., Pressman E., Meir S., Faigenboim A., Firon N. (2018). Proteomics of heat-stress and ethylene-mediated thermotolerance mechanisms in tomato pollen grains. Front. Plant Sci..

[9] Poór P., Nawaz K., Gupta R., Ashfaque F., Khan M.I.R. (2022). Ethylene involvement in the regulation of heat stress tolerance in plants. Plant Cell Rep..

[10] Singh M.B., Lohani N., Bhalla P.L. (2021). The role of endoplasmic reticulum stress response in pollen development and heat stress tolerance. Front. Plant Sci..

[11] Wu C., Tang S., Li G., Wang S., Fahad S., Ding Y. (2019). Roles of phytohormone changes in the grain yield of rice plants exposed to heat: A review. PeerJ.

[12] Yu M., Lamattina L., Spoel S.H., Loake G.J. (2014). Nitric oxide function in plant biology: A redox cue in deconvolution. New Phytol..

[13] Popova L., Tuan T. (2010). Nitric oxide in plants: Properties, biosynthesis and physiological functions. Iran. J. Sci. Technol..

[14] Mir I.R., Rather B.A., Sehar Z., Masood A., Khan N.A. (2023). Nitric oxide in co-ordination with nitrogen reverses cadmium-inhibited photosynthetic activity by interacting with ethylene synthesis, strengthening antioxidant system, and nitrogen and sulfur assimilation in mustard (*Brassica juncea* L.). Sci. Hortic..

[15] González-Gordo S., Palma J.M., Corpas F.J. (2023). Small Heat Shock Protein (sHSP) Gene Family from Sweet Pepper (*Capsicum annuum* L.) Fruits: Involvement in Ripening and Modulation by Nitric Oxide (NO). Plants.

[16] Kaya C., Ugurlar F., Ashraf M., Alam P., Ahmad P. (2023). Nitric oxide and hydrogen sulfide work together to improve tolerance to salinity stress in wheat plants by upraising the AsA-GSH cycle. Plant Physiol. Biochem..

[17] Mir I.R., Rather B.A., Masood A., Khan N.A. (2022). Nitric Oxide-and Sulfur-Mediated Reversal of Cadmium-Inhibited Photosynthetic Performance Involves Hydrogen Sulfide and Regulation of Nitrogen, Sulfur, and Antioxidant Metabolism in Mustard. Stresses.

[18] Sehar Z., Masood A., Khan N.A. (2019). Nitric oxide reverses glucose-mediated photosynthetic repression in wheat (*Triticum aestivum* L.) under salt stress. Environ. Exp. Bot..

[19] Gayatri G., Agurla S., Raghavendra A. (2013). Nitric oxide in guard cells as an important secondary messenger during stomatal closure. Front. Plant Sci..

[20] Akter N., Rafiqul Islam M. (2017). Heat stress effects and management in wheat. A review. Agron. Sustain. Dev..

[21] Lal M.K., Tiwari R.K., Gahlaut V., Mangal V., Kumar A., Singh M.P., Zinta G. (2022). Physiological and molecular insights on wheat responses to heat stress. Plant Cell Rep..

[22] Jing J., Guo S., Li Y., Li W. (2020). The alleviating effect of exogenous polyamines on heat stress susceptibility of different heat resistant wheat (*Triticum aestivum* L.) varieties. Sci. Rep..

[23] Asseng S., Ewert F., Martre P., Rötter R.P., Lobell D.B., Cammarano D., Kimball B.A., Ottman M.J., Wall G.W., White J.W. (2015). Rising temperatures reduce global wheat production. Nat. Clim. Chang..

[24] Riaz M.W., Yang L., Yousaf M.I., Sami A., Mei X.D., Shah L., Ma C. (2021). Effects of heat stress on growth, physiology of plants, yield and grain quality of different spring wheat (*Triticum aestivum* L.) genotypes. Sustainability.

[25] IPCC (2021). Climate Change 2021: The Physical Science Basis. Contribution of Working Group I to the Sixth Assessment Report of the Intergovernmental Panel on Climate Change.

[26] Liu M., Tay N.S., Bell S., Belusko M., Jacob R., Will G., Bruno F. (2016). Review on concentrating solar power plants and new developments in high temperature thermal energy storage technologies. Renew. Sustain. Energy Rev..

[27] Sarwar M., Saleem M.F., Ullah N., Rizwan M., Ali S., Shahid M.R., Ahmad P. (2018). Exogenously applied growth regulators protect the cotton crop from heat-induced injury by modulating plant defense mechanism. Sci. Rep..

[28] Chaudhary C., Sharma N., Khurana P. (2021). Decoding the wheat awn transcriptome and overexpressing Ta Rca1β in rice for heat stress tolerance. Plant Mol. Biol..

[29] Iqbal N., Umar S., Khan N.A., Corpas F.J. (2021). Nitric oxide and hydrogen sulfide coordinately reduce glucose sensitivity and decrease oxidative stress via ascorbate-glutathione cycle in heat-stressed wheat (*Triticum aestivum* L.) plants. Antioxidants.

[30] Domingos P., Prado A.M., Wong A., Gehring C., Feijo J.A. (2015). Nitric oxide: A multitasked signaling gas in plants. Mol. Plant.

[31] Bouchard J.N., Yamasaki H. (2008). Heat stress stimulates nitric oxide production in Symbiodinium microadriaticum: A possible linkage between nitric oxide and the coral bleaching phenomenon. Plant Cell Physiol..

[32] Christou A., Filippou P., Manganaris G.A., Fotopoulos V. (2014). Sodium hydrosulfide induces systemic thermotolerance to strawberry plants through transcriptional regulation of heat shock proteins and aquaporin. BMC Plant Biol..

[33] Sehar Z., Iqbal N., Khan M.I.R., Masood A., Rehman M., Hussain A., Khan N.A. (2021). Ethylene reduces glucose sensitivity and reverses photosynthetic repression through optimization of glutathione production in salt-stressed wheat (*Triticum aestivum* L.). Sci. Rep..

[34] Khan M.I.R., Iqbal N., Masood A., Per T.S., Khan N.A. (2013). Salicylic acid alleviates adverse effects of heat stress on photosynthesis through changes in proline production and ethylene formation. Plant Signal. Behav..

[35] Santisree P., Bhatnagar-Mathur P., Sharma K.K. (2015). NO to drought-multifunctional role of nitric oxide in plant drought: Do we have all the answers?. Plant Sci..

[36] Awasthi R., Bhandari K., Nayyar H. (2015). Temperature stress and redox homeostasis in agricultural crops. Front. Environ. Sci..

[37] Tian Q.Y., Sun D.H., Zhao M.G., Zhang W.H. (2007). Inhibition of nitric oxide synthase (NOS) underlies aluminum-induced inhibition of root elongation in Hibiscus moscheutos. New Phytol..

[38] Song L., Ding W., Shen J., Zhang Z., Bi Y., Zhang L. (2008). Nitric oxide mediates abscisic acid induced thermotolerance in the calluses from two ecotypes of reed under heat stress. Plant Sci..

[39] Palmieri M.C., Lindermayr C., Bauwe H., Steinhauser C., Durner J. (2010). Regulation of plant glycine decarboxylase by S-nitrosylation and glutathionylation. Plant Physiol..

[40] Parankusam S., Adimulam S.S., Bhatnagar-Mathur P., Sharma K.K. (2017). Nitric oxide (NO) in plant heat stress tolerance: Current knowledge and perspectives. Front. Plant Sci..

[41] Yang W., Sun Y., Chen S., Jiang J., Chen F., Fang W., Liu Z. (2011). The effect of exogenously applied nitric oxide on photosynthesis and antioxidant activity in heat stressed chrysanthemum. Biol. Plant..

[42] Usuda H. (1987). Changes in levels of intermediates of the C4 cycle and reductive pentose phosphate pathway under various concentrations of CO_2_ in maize leaves. Plant Physiol..

[43] Bates L.S., Waldren R.P., Teare I.D. (1973). Rapid determination of free proline for water-stress studies. Plant Soil.

[44] Grieve C.M., Grattan S.R. (1983). Rapid assay for determination of water soluble quaternary ammonium compounds. Plant Soil.

[45] Okuda T., Matsuda Y., Yamanaka A., Sagisaka S. (1991). Abrupt increase in the level of hydrogen peroxide in leaves of winter wheat is caused by cold treatment. Plant Physiol..

[46] Dhindsa R.S., Plumb-Dhindsa P.A.M.E.L.A., Thorpe T.A. (1981). Leaf senescence: Correlated with increased levels of membrane permeability and lipid peroxidation, and decreased levels of superoxide dismutase and catalase. J. Exp. Bot..

[47] Das R., Uprety D.C. (2006). Interactive effect of moisture stress and elevated CO_2_ on the oxidative stress in Brassica species. J. Food Agric. Environ..

[48] Beyer W.F., Fridovich I. (1987). Assaying for superoxide dismutase activity: Some large consequences of minor changes in conditions. Anal. Biochem..

[49] Giannopolitis C.N., Ries S.K. (1977). Superoxide dismutases: I. Occurrence in higher plants. Plant Physiol..

[50] Nakano Y., Asada K. (1981). Hydrogen peroxide is scavenged by ascorbate-specific peroxidase in spinach chloroplasts. Plant Cell Physiol..

[51] Foyer C.H., Halliwell B. (1976). The presence of glutathione and glutathione reductase in chloroplasts: A proposed role in ascorbic acid metabolism. Planta.

[52] Zhou B., Guo Z., Xing J., Huang B. (2005). Nitric oxide is involved in abscisic acid-induced antioxidant activities in *Stylosanthes guianensis*. J. Exp. Bot..

[53] Avni A., Bailey B.A., Mattoo A.K., Anderson J.D. (1994). Induction of ethylene biosynthesis in Nicotiana tabacum by a Trichoderma viride xylanase is correlated to the accumulation of 1-aminocyclopropane-1-carboxylic acid (ACC) synthase and ACC oxidase transcripts. Plant Physiol..

[54] Woeste K.E., Ye C., Kieber J.J. (1999). Two Arabidopsis mutants that overproduce ethylene are affected in the posttranscriptional regulation of 1-aminocyclopropane-1-carboxylic acid synthase. Plant Physiol..

[55] Fatma M., Iqbal N., Gautam H., Sehar Z., Sofo A., D’Ippolito I., Khan N.A. (2021). Ethylene and sulfur coordinately modulate the antioxidant system and ABA accumulation in mustard plants under salt stress. Plants.

[56] Turano F.J., Thakkar S.S., Fang T., Weisemann J.M. (1997). Characterization and expression of NAD (H)-dependent glutamate dehydrogenase genes in Arabidopsis. Plant Physiol..

