# Supplementary material for: Nitric Oxide and Proline Modulate Redox Homeostasis and Photosynthetic Metabolism in Wheat Plants under High Temperature Stress Acclimation
Source: Plants (Basel). 2023 Mar 10;12(6):1256. doi: 10.3390/plants12061256 (PMC10053195; doi:10.3390/plants12061256)
Supplement: Supplementary file 1 [file plants-12-01256-s001.zip › Supplementary file.pdf]

Supplementary File S1: Methodology and Table S1

Article

Nitric oxide and proline modulate redox homeostasis and photosynthetic metabolism in wheat plants under high temperature stress acclimation

Zebus Sehar, Iqbal R. Mir, Sheen Khan, Asim Masood and Nafees A. Khan*

Plant Physiology and Biochemistry Laboratory, Department of Botany, Aligarh Muslim University, Aligarh-202002, India

Corresponding author: naf9.amu@gmail.com;

Assay of Antioxidant Enzymes

Fresh leaves (200 mg) were homogenized with an extraction buffer containing 0.05% (v/v) Triton X-100 and 1% (w/v) PVP in potassium-phosphate buffer (100 mM, pH 7.0) using chilled mortar and pestle. The homogenate was centrifuged at 15,000 x g for 20 min at 4oC. The supernatant obtained after centrifugation was used for the assay of SOD (EC; 1.15.1.1) and GR (EC; 1.6.4.2) enzymes. For the assay of APX (EC; 1.11.1.11), 2.0 mM ascorbate was supplemented with extraction buffer.

Activity of SOD was determined by adopting the methods of Beyer and Fridovich (1987) and Giannopolitis and Ries (1977) by monitoring the inhibition of photochemical reduction of NBT. 5 mL of reaction mixture consisting of 5 mM HEPES (pH 7.6), 50 mM Na2CO3 (pH 10.0), 0.1 mM EDTA, 0.025% (v/v) Triton X-100, 13 mM methionine, 63 mmol NBT and 1.3 mmol of riboflavin. The enzyme extract was Illuminated for 15 min (360 μmol m^2s^{-1}), and a set was not illuminated which acted as a control to correct for the turbidity of background absorbance. One unit of SOD is defined as the amount of enzyme that inhibited the NBT reduction by 50% at 560 nm. Amount of enzyme that inhibited the reduction of NBT by 50 % at 560 nm is equal to one unit of SOD.

The activity of APX was determined following the method of Nakano and Asada (1981) by recording the decrease in the absorbance of ascorbate at 290 nm. A 1.0 mL assay mixture contained 50 mM phosphate buffer (pH 7.0), 0.1 mM EDTA, 0.5 mM ascorbate and 0.1 mM H_2O_2, and the enzyme extract. APX activity was calculated by using the extinction coefficient of 2.8 $mM^{-1}cm^{-1}$. One unit of the enzyme is the amount necessary to decompose 1 μmol of substrate per min at 25°C.

The activity of GR was determined by the method of Foyer and Halliwell (1976) by monitoring the glutathione-dependent oxidation of NADPH at 340 nm. The reaction mixture contained phosphate buffer (25 mM, pH 7.8), 0.5 mM GSSG, 0.2mM NADPH, and the enzyme extract. The activity of GR was calculated by using the extinction coefficient 6.2 mM-1 cm-1. One unit of enzyme is the amount necessary to decompose 1 μmol of NADPH per min at 25°C.

Determination of H_2O_2 Content and Lipid Peroxidation

H_2O_2 content in leaf tissues (500 mg) was kept in ice-cold 200 mM $HClO_4$ was measured using the Okuda et al. (1991) technique. This was centrifuged at 1200 g for 10 minutes followed by the neutralization of $HClO_4$ of the supernatant with 4 M KOH. Further centrifugation at 500 g for 3 minutes removed the insoluble $KClO_4$. The reaction mixture contains 1 ml of the eluate, 400 μl of 12.5 mM 3-(dimethylamino) benzoic acid in 0.375 M phosphate buffer (pH 6.5), 80μl of 3-methyl-2-

benzothiazoline hydrazone, and 20µl of peroxidase in a final volume of 1.5 ml (0.25 Unit). At 25°C, peroxidase was added to initiate the reaction, and the absorbance was obtained at 590 nm.

The concentration of thiobarbituric acid reactive substances (TBARS) was calculated using the Dhindsa et al. (1981) technique to evaluate lipid peroxidation or membrane damage. Leaf tissues (500 mg) were powdered in 0.25 % 2-thiobarbituric acid in 10% trichloroacetic acid, then heated for 30 minutes at 95°C and cooled fast on an ice bath. After that, the samples were centrifuged for 10 minutes at 10,000 g. A 1 ml sample of the supernatant was treated with 4 ml of 20% trichloroacetic acid containing 5% thiobarbituric acid. At 532 nm, the colour intensity was measured.

RNA Isolation and cDNA Synthesis

Total RNA was isolated from rice leaves using TRIzol reagent (Ambion, Life Technologies, USA) according to the manufacturer's instructions. With the help of a Nanodrop spectrophotometer (Thermo Scientific, USA), the extracted RNA was quantified. To ensure the integrity of the RNA, each sample was run on agarose formaldehyde gel Turano et al. (1997). The first-strand of the cDNA was made from 1 µg of total RNA of control and treated samples. The cDNA template was synthesized using the reaction mixture containing 20 U/µL Moloney murine leukemia virus reverse transcriptase (MuMLV) enzyme (Fermentas, USA) at 42°C for 50 min and at 70°C for 10 min. The reverse transcription reaction was carried out using 2.5 µM Oligo (dT) 18 primer (Fermentas, USA) and 10 mM dNTPs. Primers for gene expression analysis were designed using online primer designing software (IDT) and cDNA sequences of selected genes were obtained from NCBI.

Quantitative Real-Time PCR Analysis

Real-time PCR (RT-PCR) was performed in 96-well reaction plate (Roche, Germany) containing 20 µL reaction mixture of × 10 reaction buffer, 2 mM dNTPs, 1 mM $MgCl_2$, 0.35 µM each of forward and reverse primers, 1 µL Sybr green (×10), 10 µg cDNA template and 5 U Taq polymerase on a thermal cycler (Light cycler 480 II, Roche, Germany). All quantifications were normalized to actin DNA fragment amplified by β-actin forward and β-actin reverse primers. The actin gene was used as an internal control for evaluating the efficiency of RT-PCR for genes. PCR cycling conditions were as follows: denaturation at 95 °C for 3 min, 40 cycles of 95°C (20 s), 66 °C (1 min) and 72 °C (1 min) with 5 min of final extension at 72 °C. The amplified product was resolved on 1.2% agarose gel. The specificity of amplicons was verified by melting curve analysis (60 to 95 °C) after 40 cycles. All reactions were performed in three biological replicates (with three technical replicates of each), using gene-specific primers and actin primers as an internal control. The data were taken as the expression of the gene of interest in relation to the internal control in the treated sample compared with the untreated control.

Reference

1. Beyer Jr, W.F.; Fridovich, I. Assaying for superoxide dismutase activity: some large consequences of minor changes in conditions. *Anal. Biochem.* **1987**, *161*, 559-566.
2. Giannopolitis, C.N.; Ries, S.K. Superoxide dismutases: I. Occurrence in higher plants. *Plant Physiol.* **1977**, *59*, 309-314.
3. Nakano, Y.; Asada, K. Hydrogen peroxide is scavenged by ascorbate-specific peroxidase in spinach chloroplasts. *Plant Cell Physiol.* **1981**, 22, 867-880.
4. Foyer, C.H.; Halliwell, B. The presence of glutathione and glutathione reductase in chloroplasts: a proposed role in ascorbic acid metabolism. *Planta* **1976**, *133*, 21-25.

5. Turano, F.J.; Thakkar, S.S.; Fang, T.; Weisemann, J.M. Characterization and expression of NAD (H)-dependent glutamate dehydrogenase genes in Arabidopsis. *Plant Physiol.* **1997**, *113*, 1329-1341.
6. Okuda, T.; Matsuda, Y.; Yamanaka, A.; Sagisaka, S. Abrupt increase in the level of hydrogen peroxide in leaves of winter wheat is caused by cold treatment. *Plant Physiol.* **1991,** 97, 1265-1267.
7. Dhindsa, R.S.; Plumb-Dhindsa, P.A.M.E.L.A.; Thorpe, T.A. Leaf senescence: correlated with increased levels of membrane permeability and lipid peroxidation, and decreased levels of superoxide dismutase and catalase. *J. Exp. Bot.* **1981**, 32, 93-101.

Table S1. Primer pairs used for quantitative RT-PCR

S.No.	Gene	Gene ID	Forward primer	Reverse primer
2.	*GR*	123146096	GCCATGTGTGGACCAGATGC	GCAGAAGGGTGGATCCCGAC
3.	*PsbA*	803183	ATATTGTGGCCGCTCAT	TCCGTTTAGATTGAAAGCCA
4.	*PsbB*	803181	GCCGGAACTATGTGGTAT	GACCAAGCTTCTGATAAAC
Reference gene primer sequences used for quantitative RT-PCR				
1.	*Actin*	123048645	GACTGCCAAGACCAGCTCC	CTTCCTAATATCCACGTCGCAC
